# Formulation and characterization of agar based aerogels as potential implantable controlled drug delivery system 

**DOI:** 10.3389/fphar.2026.1752045

**Published:** 2026-03-30

**Authors:** Shaker T. Alsharif

**Affiliations:** Pharmaceutical Science Department, College of Pharmacy, Umm Al-Qura University, Makkah, Saudi Arabia

**Keywords:** agar aerogels, caffeine release, drug delivery systems, HPLC, localized implants, mechanical strength, porosity, post-loading

## Abstract

**Introduction:**

Agar-based aerogels have emerged as promising candidates for implantable drug delivery systems due to their tunable porosity, mechanical properties, and biocompatibility.

**Methods:**

Agar aerogels were prepared at varying concentrations (2, 2.5, 3, and 3.5% w/v) and loaded with caffeine as a model drug using preloading and post-loading techniques. The formulations were characterized for mechanical strength, porosity, swelling behavior, drug content distribution, and in vitro release profiles. Raman mapping and HPLC analysis were employed to evaluate drug distribution and quantification.

**Results:**

Increasing agar concentration significantly enhanced mechanical strength while reducing porosity and swelling capacity. Preloaded aerogels demonstrated improved mechanical stability, whereas post-loaded aerogels exhibited reduced strength with increasing loading volume. Drug distribution was more homogeneous at lower agar concentrations and moderate drug loadings. All formulations exhibited biphasic release profiles, with an initial burst release followed by sustained release, influenced by agar concentration, drug loading, and loading method.

**Discussion:**

Optimized formulations, particularly preloaded aerogels with 5% caffeine and post-loaded aerogels with 1% caffeine at controlled volumes, demonstrated desirable mechanical and release characteristics. These findings highlight the potential of agar-based aerogels as versatile and tunable systems for localized controlled drug delivery.

## 1 Introduction

Aerogels are defined as a highly porous structured matrix system with interesting features making them attractive to be investigated as a potential drug delivery system. These features include significant low density, high surface area, and high porosity (Du et al., 2013). Also, A wide range of biomaterials can be used for aerogels development comprise, the uncomplicated conditions required for their preparations. Furthermore, the versatility of tuning aerogels mesh size, interconnected pores, and shapes are promising properties to explore aerogels capabilities as pharmaceutical formulations (Barrios et al., 2019; Liu et al., 2020). In comparison to common hydrogels, their freeze-dried aerogels may demonstrate faster loading of drug molecules, easier access to core region of the matrix, and more efficient interactions with the aerogel matrix. Nevertheless, adjusting the aerogels polymeric components and the rate of crosslinking, could influence aerogels’ ability to control drug release or, in contrast, their ability as drug diffusing enhancers (Valo et al., 2013; Alnaief et al., 2012; Ulker and Erkey, 2014; Veronovski et al., 2013).

In addition, the adjustable porous flexible mechanical structure of aerogels could make them capable to be designed into different shapes and sizes, which is helpful to develop the aerogels as a local drug delivery system. Also, aerogels could offer the option for drug loading method to be either preloaded during the formulation preparation or post-loaded after obtaining the formulation. The post loading method may offer better flexibility for the surgical team to load the required therapeutic agents and dose upon implantation.

Several naturally sourced polysaccharide biopolymers for the development of aerogel formulations have been shown to be superior in terms of mechanical properties, sustainability, and biocompatibility (Wei et al., 2020; Nita et al., 2020). This chapter will focus on the assessment of highly porous agar matrices as aerogels encapsulating caffeine as a model drug. The main goals are to investigate the effect of varying the agar concentration (2, 2.5, 3% and 3.5%), caffeine loading (1, 5% and 10%), loading method on the mechanical properties of the aerogels, as well as the distribution of caffeine within the aerogels and its subsequent release.

Although previous studies have examined the influence of polymer concentration or drug loading on aerogel properties (Zhao et al., 2024), a comprehensive evaluation integrating agar concentration, loading strategy (pre-loading versus post-loading), and multi-level drug content within a unified experimental framework remains limited. In particular, the combined assessment of mechanical behavior, spatial drug distribution, and release kinetics under controlled compositional variation has not been systematically reported for agar-based aerogels. Therefore, the present study aims to bridge this gap by establishing structure property release relationships that are directly relevant to implantable controlled drug delivery systems.

## 2 Materials and methods

### 2.1 Materials

A polysaccharide complex Agar composed of about 70% agarose and 30% agaropectin (Sigma-Aldrich; Cat no. A9799; high gel strength powder; CAS 9002-18-0) was used for hydrogel preparation. Caffeine (Thermo Scientific Alfa Aesar Chemicals; Cat no.11428643, 1,000 g pack; assay ≥99%; CAS 58-08-2) was used as the model drug. Methanol (Fisher Scientific; HPLC grade, 99.9%) and phosphate-buffered saline (PBS) tablets (VWR, United Kingdom) were used as received.

### 2.2 Preparation of caffeine preloaded and post-loaded agar aerogels

The agar aerogels were prepared using predetermined amounts (Table 1) of agar in distilled water to prepare four different hydrogel formulations with agar concentrations of 2, 2.5, 3 and 3.5% w/v. predetermined amounts of caffeine were added to the mixtures. The final agar hydrogel formulation batches contained either 1, 5, and 10% w/v of caffeine. To prepare the caffeine preloaded aerogels, the obtained hydrogels were frozen at −80 °C for 48 h to ensure complete solidification. The frozen samples were subsequently lyophilized using a Zirbus VaCo 10 laboratory freeze dryer (Zirbus Technology GmbH, Germany) equipped with a stainless-steel chamber. Freeze-drying was conducted under reduced pressure (∼0.05–0.1 mbar) for 48 h to allow sublimation of ice and removal of residual moisture. The condenser temperature was maintained at −80 °C throughout the drying process. No programmed shelf temperature ramp was applied. Alternatively, post-loaded aerogels were prepared using the same method without prior loading of caffeine as blank aerogels only. The caffeine post loading was performed by injecting different volumes (1, 2, and 3 mL) from different caffeine concentrations stock solutions (1, 5, and 10% w/v) (Table 2) to evaluate the effect of volume, concentration, and loading method.

**TABLE 1:** Formulations of prepared caffeine loaded hydrogels.

Formulations	Agar % w/v	Caffeine % w/v	Hydrogel solution volume mL
1	2	1	10
2	2	5	10
3	2	10	10
4	2.5	1	10
5	2.5	5	10
6	2.5	10	10
7	3	1	10
8	3	5	10
9	3	10	10
10	3.5	1	10
11	3.5	5	10
12	3.5	10	10

**TABLE 2:** Formulations of prepared caffeine post-loaded aerogels.

Agar aerogel % w/v	Caffeine solution % w/v	Loading volume mL
2	1	1, 2, 3
	5	1, 2, 3
	10	1, 2, 3
2.5	1	1, 2, 3
	5	1, 2, 3
	10	1, 2, 3
3	1	1, 2, 3
	5	1, 2, 3
	10	1, 2, 3
3.5	1	1, 2, 3
	5	1, 2, 3
	10	1, 2, 3

### 2.3 Characterization of the agar aerogel formulations

#### 2.3.1 Aerogels mechanical strength

The effects of agar concentration and loading methods on the strength of the aerogels was evaluated. Experiments were performed on different agar aerogels concentrations of 2, 2.5, 3, and 3.5% without drug loading as well as 2 and 2.5% agar hydrogels preloaded with 1 and 5% caffeine, while 3 and 3.5% agar hydrogels preloaded with 1% caffeine. Also, the impact of post-loading method was investigated by injecting different volumes of 1, 2, and 3 mL of deionized water into each formulation. The mechanical strength of the samples was evaluated using a texture analyzer (TA. XT Plus C) fitted with a 20 mm cylindrical probe and coupled with an oven to maintain a controlled temperature of 37 °C. The aerogels were positioned beneath the probe, which was operated under a maximum applied force of 5 kg at a constant crosshead speed of 1 mm/s to a penetration depth of 10 mm. A penetration depth of 10 mm was selected to allow sufficient deformation of the aerogel matrix to assess compressive resistance while avoiding complete structural collapse, thereby simulating mechanical stresses relevant to potential implantation conditions. Mechanical property assessments were carried out in triplicate on independent formulation batches, with drug-free aerogels employed as controls.

#### 2.3.2 Aerogels porosity and swelling ratios

The porosity and swelling ratios of the aerogels were evaluated using different agar aerogel concentrations of 2, 2.5, 3, and 3.5%. First, the porosity tests were performed using liquid the displacement method. Every aerogel was placed into 30 mL of ethanol (V_1_) inside a 50 mL centrifuge tube. The volume of ethanol with the aerogel was recorded (V_2_). Then, the aerogel was removed from the centrifuge tube, and the remaining volume was recorded (V_3_). The porosity ratio (*ε*1) was calculated using Equation 1 (Kanokpanont et al., 2012). The aerogel swelling ratio was determined by measuring the weight of each dry aerogel (W_0_). Every aerogel was submerged into a bath of deionized water for 1 h at room temperature. After that, aerogels were removed from the water bath and excess water on the surface was removed via filter paper. The aerogels weight was then measured again (W_1_). Equation 2 was used to determine the swelling ratio (*ε2*) (Yang et al., 2018). The porosity and swelling ratios of the aerogels were performed in triplicate using separate formulation batches.

$$\varepsilon 1\;\left(\%\right)=\left(V1-V3\right)\;/\;\left(V2-V3\right)\times 100$$ (1)

$$\varepsilon 2\;\left(\%\right)=\left(W1-W0\right)\;/\;W0\times 100$$ (2)

#### 2.3.3 Raman mapping of caffeine post-loaded aerogels

The aim of this test is to determine the spatial distribution of caffeine post-loaded agar aerogels. The aerogels were investigated by collecting approximately 5 × 3 mm dried samples from the 2 and 2.5% aerogels post-loaded with 1 mL of different caffeine concentrations 1, 5, and 10%. The samples were placed and fixed on microscopic glass slides using double sided tape. Raman mapping tests were performed using a Thermo Scientific™ DXR system fitted with an external DXR 720 nm frequency laser source and an Olympus TH4-200 optical microscope. A ×10 objective was used for optical imaging and spectrum acquisition. All spectra were obtained in the spectral range of 200–3,413 cm^−1^ with a resolution range 2.4–4.4 cm^−1^ provided by a grating monochromator of 400 lines/mm. Raman maps and reference spectra of the samples were collected using laser spot size 3.1 μm and 30 μm step size. The reference Raman spectra of the pure caffeine was collected in a powder form. The measured area for every sample was 0.4 mm × 0.4 mm.

#### 2.3.4 Caffeine content distribution of the preloaded aerogel formulations

The caffeine preloaded agar aerogels were divided into three equal portions and placed in glass bottles containing 100 mL of a 50:50 (v/v) methanol–deionized water mixture. The samples were subjected to vigorous stirring overnight and subsequently vortexed to ensure exhaustive extraction of caffeine. Aliquots of 1 mL from each extract were centrifuged at 2,100 *g* for 15 min, passed through a 0.45 μm membrane filter, and analyzed by HPLC.

#### 2.3.5 *In vitro* release of caffeine from pre- and post-loaded aerogels

0.4–1.5 g (dependent on their agar and caffeine content) of the preloaded agar aerogels were placed into a glass bottle containing 100 mL of pre-prepared PBS buffer (pH 7.4). The post-loaded aerogels were fixed one side so that they remained in floating position in plastic tubes containing 50 mL of pre-prepared PBS buffer (pH 7.4) and placed in an orbital shaking incubator at 37 °C and 60 rpm. The aerogels were fixed by pushing a needle through the plastic cap of the tube and into the aerogel. At time intervals of 0, 1, 3, 6, 24, and 48 h a 1 mL sample was collected, replaced with 1 mL of fresh PBS buffer, and subsequently stored at 4 °C until analysed by HPLC. The release study was conducted in triplicate and the percentage of caffeine released calculated relative to the caffeine content of the aerogels.

### 2.4 HPLC analysis

#### 2.4.1 HPLC chromatographic conditions

The caffeine was analysed using an Agilent HPLC System (Agilent Technologies 1,260 infinity II, Santa Clara, California, United States) equipped with a quadratic pump and autosampler. The separation was performed using a Thermo Scientific® 5 μm, C18 analytical HPLC column (150 mm *4.6 cm) at 25 °C with a mobile phase composed of methanol and deionized water in 50: 50 ratios at a flow rate of 1 mL/min. An injection volume of 10 μL and run time of 10 min was used, and UV detection was conducted at 273 nm.

#### 2.4.2 Stock solution and calibration curve

A caffeine stock solution (1 mg/mL) was prepared in 50:50 (v/v) methanol–deionized water and diluted to obtain working standards of 0.001–1 mg/mL. Calibration curves were generated by plotting peak area versus concentration. All measurements were performed in triplicate.

#### 2.4.3 Method validation

The HPLC method was validated for linearity, sensitivity, and precision in accordance with standard analytical validation practices. Calibration curves were constructed using caffeine standards in the concentration range of 0.001–1 mg/mL. Each concentration level was analyzed in triplicate (n = 3), and calibration was performed by plotting mean peak area versus concentration. Linearity was evaluated by least-squares linear regression analysis. The limit of detection (LOD) and limit of quantification (LOQ) were calculated using the standard deviation of the residuals (σ) and the slope of the calibration curve (S), according to the equations:

LOD=3.3σ/S

LOQ=10σ/S

Intra-day precision was assessed by calculating the relative standard deviation (RSD%) of triplicate measurements at each concentration level.

### 2.5 Statistical analysis

Statistical analyses were conducted using one-way or two-way analysis of variance (ANOVA), depending on the number of independent variables, with GraphPad Prism (version 9.0.2, MacOS; GraphPad Software, San Diego, CA). Post hoc comparisons were performed using Tukey’s Honestly Significant Difference test, and statistical significance was defined as *p* < 0.05.

## 3 Results

### 3.1 Aerogels mechanical properties

**FIGURE 1:**
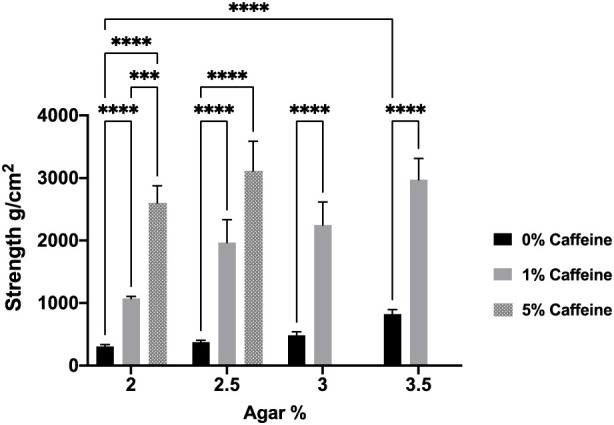
Effect of different agar concentrations (2, 2.5, 3, and 3.5%) and caffeine loading concentrations (1%and 5%) aerogels mechanical strength. Results shown as mean ± SD, n = 3, significance found using a two-way ANOVA with a Tukey’s *post hoc* test, * = *p* < 0.05 ** = *p* < 0.01, *** = *p* < 0.001, **** = *p* < 0.0001.

**FIGURE 2:**
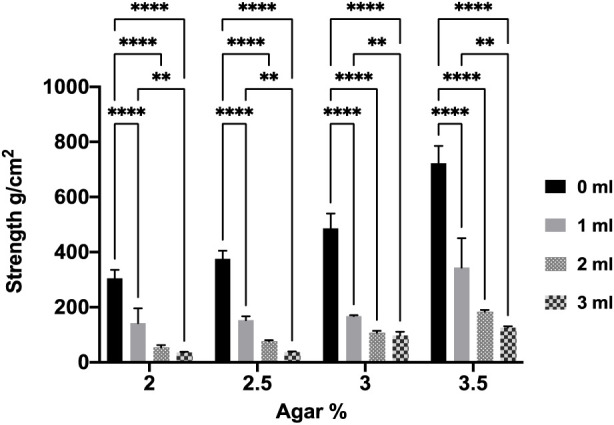
Effect of post loading volumes (1, 2, and 3 mL) on 2, 2.5, 3, and 3.5% agar aerogels mechanical strength. Results shown as mean ± SD, n = 3, significance found using a two-way ANOVA with a Tukey’s *post hoc* test, * = *p* < 0.05 ** = *p* < 0.01, *** = *p* < 0.001, **** = *p* < 0.0001.

The mechanical tests performed on the agar aerogels demonstrated that the higher agar concentration in the unloaded aerogels significantly increased their mechanical strength (Figure 1). Results in Figure 1 report that the mechanical strength levels recorded were significantly (*p* < 0.05) increased from 305.5 ± 31 g/cm^2^ for the 2% agar aerogel to 375.8 ± 30 g/cm^2^ with the 2.5% agar aerogel, while the 3 and 3.5% agar aerogels showed a significant increase in their physical strength increasing to 486.8 ± 54 and 822.1 ± 64 g/cm^2^, respectively. Nevertheless, the agar aerogel formulations preloaded with either 1 or 5% caffeine demonstrated improved mechanical properties compared to the unloaded aerogels. Moreover, increasing the caffeine loading provided a further significant increase in their mechanical strength. However, the post-loaded agar aerogels demonstrated a significant reduction in their measured strength levels upon post loading, which further reduced with increasing injection volume (Figure 2).

### 3.2 Aerogel porosity and swelling ratios

**FIGURE 3:**
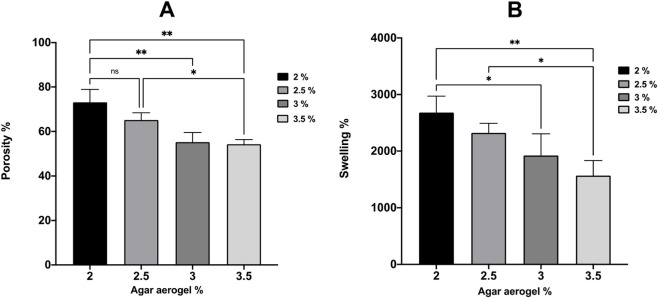
Effect of varying agar concentrations (2, 2.5, 3, and 3.5%) on aerogels porosity **(A)**, and swelling **(B)**. Results shown as mean ± SD, n = 3, significance found using a one-way ANOVA with a Tukey’s *post hoc* test, * = *p* < 0.05 ** = *p* < 0.01, *** = *p* < 0.001, **** = *p* < 0.0001.

The mean porosity and swelling ratios for the agar aerogels demonstrated a significant decrease in both porosity and swelling as the agar concentration increased (Figure 3). The porosity ratios of agar aerogels showed a non-significant reduction from 73.1% ± 5.7% to 65.2% ± 3.2% as the agar aerogel concentration increased from 2% to 2.5% (Figure 3A). However, the porosity ratio of both 3 and 3.5% agar aerogels demonstrated a significant reduction (55.2% ± 4.3% and 54.3% ± 2.1%, respectively) compared to 2 and 2.5% aerogels. Their swelling ratios demonstrated a non-significant reduction from 2,681.9% ± 291.9% to 2,322.9% ± 169.9% for 2 and 2.5% aerogels, respectively. However, a significant reduction in the swelling ratios was observed with the 3 and 3.5% aerogels at 1922.7% ± 383.8%, and 1,568.6% ± 265% respectively as seen in Figure 3B.

### 3.3 Raman mapping of the caffeine post-loaded aerogels

**FIGURE 4:**
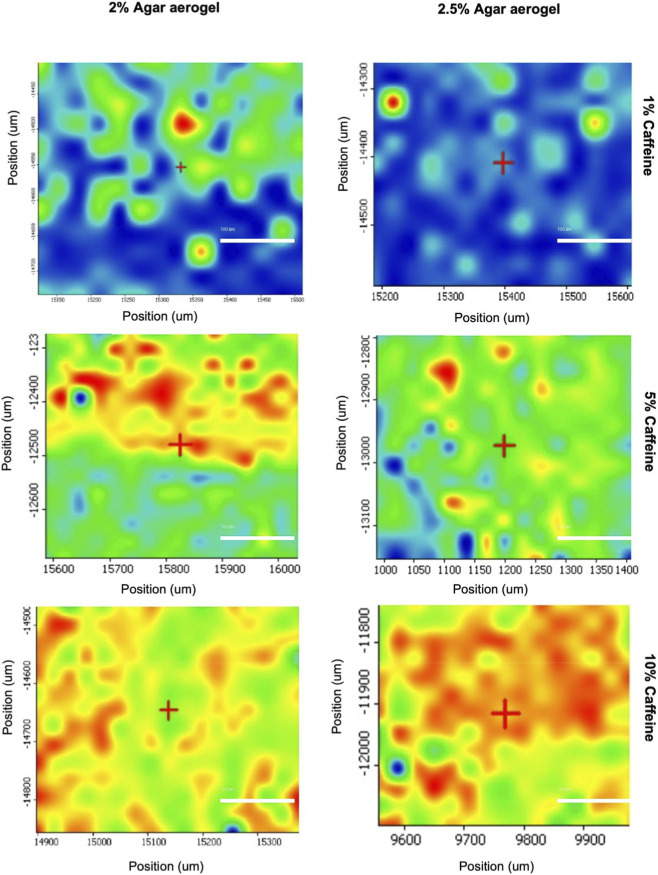
Raman distribution maps of 2% and 2.5% agar aerogels post-loaded with 1 mL of different caffeine concentrations (1, 5, and 10%). Mapping was performed over a 0.4 mm × 0.4 mm area using a 30 µm step size and a 3.1 µm laser spot size. Scale bar represents 100 µm.

**FIGURE 5:**
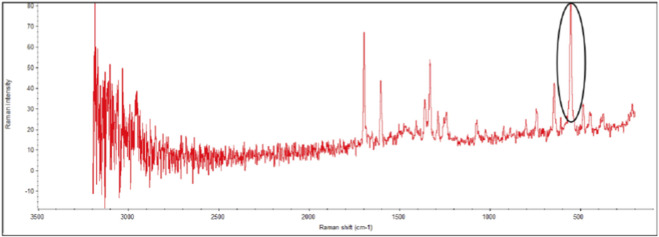
The reference Raman spectra of the pure caffeine.

The distribution Raman maps of the 2 and 2.5% agar aerogels post-loaded with caffeine (1, 5, and 10%) are presented in Figure 4, with the reference Raman spectra of the pure caffeine presented in Figure 5. Caffeine distribution is highlighted using the following colour scheme; no caffeine (Blue), low caffeine distribution (Green), medium caffeine distribution (Yellow) and high caffeine distribution (Red). Results showed that the aerogels caffeine content distribution within the aerogel samples was varied based on the agar concentration and the caffeine loading rate. The higher the agar concentration and the lower the caffeine concentration of the solution are related to the lower distribution of the caffeine in the aerogels. However, with the higher concentration caffeine solutions the distribution is significantly improved. This is demonstrated by the significant levels of blue (no caffeine) in both the 2 and 2.5% agar aerogels with the 1% caffeine solutions, while for the 5% and 10% caffeine solution the blue disappears and there is an increase in green (low caffeine distribution), yellow (medium caffeine distribution) and red (high caffeine distribution).

### 3.4 HPLC analysis of caffeine

**FIGURE 6:**
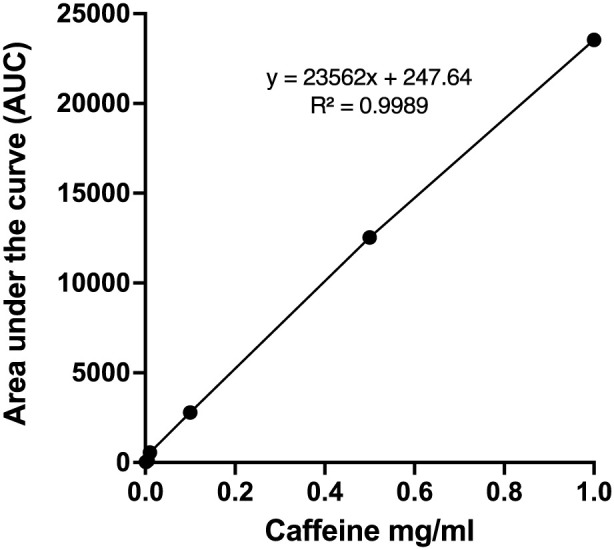
Standard curve for caffeine using HPLC (n = 3).

**FIGURE 7:**
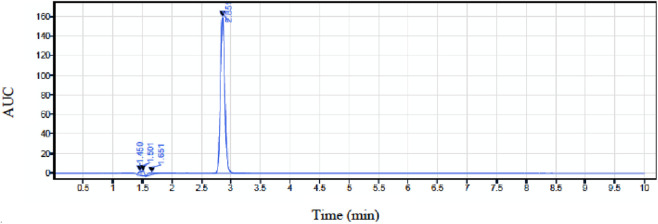
The HPLC chromatogram depicting the peak of caffeine at 273 nm wavelength.

The calibration curve for caffeine exhibited excellent linearity over the concentration range of 0.001–1 mg/mL. Linear regression analysis yielded the following equation:

Peak Area=23562C+247.64

with a correlation coefficient (*R* ^2^) of 0.9989, indicating strong linear correlation between concentration and peak area (Figure 6). The calculated limit of detection (LOD) and limit of quantification (LOQ) were 0.0487 mg/mL and 0.1476 mg/mL, respectively. Triplicate injections demonstrated good repeatability, with relative standard deviation (RSD) values below 2% across the tested concentration range. The retention time of caffeine was approximately 2.9 min, as shown in Figure 7. These results confirm that the analytical method is sufficiently sensitive, precise, and reliable for quantification of caffeine in the release studies.

### 3.5 Caffeine content distribution of preloaded agar aerogel formulations

**FIGURE 8:**
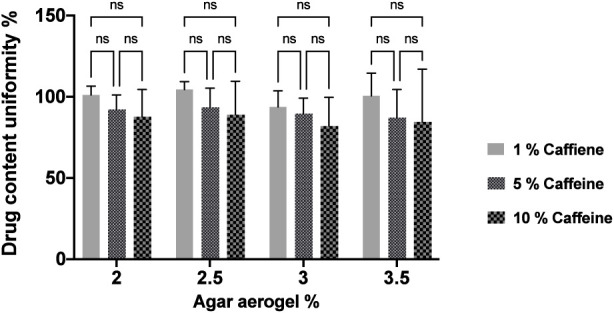
Caffeine content distribution (1, 5, and 10%) in four preloaded aerogels (2, 2.5, 3, and 3.5%). Results shown as mean ± SD, significance found using a two-way ANOVA with a Tukey’s *post hoc* test, * = *p* < 0.05 ** = *p* < 0.01, *** = *p* < 0.001, **** = *p* < 0.0001.

The caffeine content distribution in agar aerogels (2, 2.5, 3, and 3.5%) preloaded with caffeine (1, 5, and 10%) was investigated. The caffeine contents distribution in preloaded aerogels varied based on the increased agar concentration and the caffeine loadings within every aerogel. As demonstrated by Figure 8, the caffeine content distribution varied with an increase in drug loading and agar concentration. The lower agar aerogels (2% and 2.5%) preloaded with 1 and 5% caffeine expressed relatively better distribution of caffeine within the aerogels compared to those with an increased agar concentration (3% and 3.5%). In addition, a reduced homogeneity was observed with an increase in caffeine loading to 10%. The 2% agar aerogel caffeine contents varied from 101.1% ± 5.5% to 87.7% ± 16.8%, while the 2.5% aerogels had caffeine contents of 104.4 ± 4.9, 93.6 ± 4.7, and 88.03% ± 20.5% for 1, 5, and 10% caffeine loading respectively. The 3% aerogels showed contents of 94.7 ± 9.8, 89.7 ± 3.8, and 82.08% ± 17.5% respectively, for the 1, 5, and 10% caffeine loadings, while the caffeine contents within 3.5% aerogels varied from 100.6% ± 13.9% to 84.9% ± 32.3% across the same caffeine loadings.

### 3.6 *In vitro* release of caffeine from preloaded agar aerogels

**FIGURE 9:**
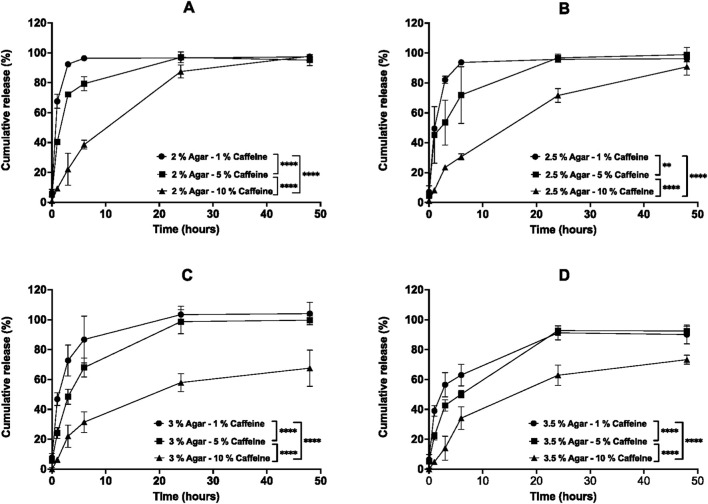
*In vitro* cumulative release profiles of caffeine (1%, 5%, and 10%) from preloaded agar aerogels: **(A)** 2%, **(B)** 2.5%, **(C)** 3%, and **(D)** 3.5% agar concentrations in PBS (pH 7.4) over 48 h at 37 °C and 60 rpm. Results are presented as mean ± SD (n = 3). Statistical significance was determined using two-way ANOVA with Tukey’s post hoc test (*p < 0.05, **p < 0.01, ***p < 0.001, ***p < 0.0001).

The release of caffeine from preloaded agar aerogels was performed in PBS (pH 7.4) at 37 °C. The percentage cumulative release profiles are presented in Figure 9. The release profiles of caffeine from the preloaded aerogels were varying based on two factors, including the increased agar concentration of the aerogels and the varying caffeine loadings within every aerogel. As seen in Figure 9, the caffeine displayed a biphasic release profile over 48 h from aerogels. First, a rapid ‘burst’ release was observed, followed by the second phase of release that yielded a slower release after 6 h as the caffeine loading, and agar concentration increased. The 2% agar preloaded aerogel loaded with 1% caffeine showed the fastest release behaviour in comparison to the same aerogels loaded with 5% and 10% caffeine (Figure 9A). Within 6 hours, the 2% aerogels released 96.3% ± 0.5% of its caffeine, while for both 5% and 10% loaded caffeine aerogels demonstrated slower release of 79.3% ± 4.7% and 38.1% ± 2.9% respectively. Most of the caffeine in the 2% aerogels were released after 48 h. Moreover, the higher agar concentration in the 2.5% aerogels resulted in slower release for 1 and 5% loaded caffeine at 3 h. The 10% caffeine preloaded aerogel showed a further significant (*p* < 0.05) reduction in its release compared to the 1 and 5% caffeine loadings within the same 2.5% agar aerogel (Figure 9B). In addition, both 3 and 3.5% aerogels reduced the initial burst release of their 1 and 5% caffeine compared previous aerogels within 3 and 6 h for 3 and 3.5% aerogels respectively. This is followed by releasing most of their contents after 48 h. Nevertheless, 10% caffeine preloaded 3 and 3.5% aerogels provided significant (*p* < 0.05) slower sustained release up to 48 h releasing a total of 67.5% ± 12.1% and 73.2% ± 3% of their caffeine content respectively as illustrated in Figures 9C,D. To facilitate comparison between formulations, key kinetic parameters including burst release (6 h), time to 50% release (t_50_%), and Korsmeyer–Peppas diffusion exponent (n) are summarized in Table 3. Formulations exhibiting rapid release reached >60% drug release before sufficient early time points were available for reliable diffusion modeling.

**TABLE 3:** Summary of release kinetics for preloaded agar aerogels.

Agar (%)	Caffeine (%)	Burst release at 6 h (% of M ∞ )	t_50_% (h)	K–P n	R^2^
2%	1%	99.0	0.69	—	—
2%	5%	83.4	1.45	—	—
2%	10%	39.5	9.78	0.804	1.000
2.5%	1%	97.5	0.97	—	—
2.5%	5%	72.7	2.02	0.153	1.000
2.5%	10%	33.7	12.48	0.756	0.960
3%	1%	83.4	1.40	—	—
3%	5%	68.2	3.21	0.632	1.000
3%	10%	46.6	7.56	0.919	0.968
3.5%	1%	69.9	1.69	—	—
3.5%	5%	54.3	4.43	0.471	0.958
3.5%	10%	46.6	7.55	1.079	0.994

### 3.7 *In vitro* release of caffeine from post-loaded aerogels

**FIGURE 10:**
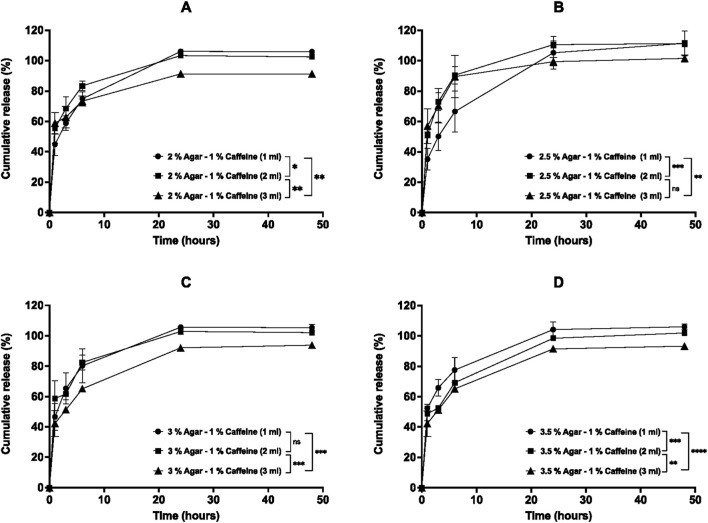
*In vitro* cumulative release profiles of 1% caffeine post-loaded at different volumes (1, 2, and 3 mL) into agar aerogels: **(A)** 2%, **(B)** 2.5%, **(C)** 3%, and **(D)** 3.5% agar concentrations in PBS (pH 7.4) over 48 h at 37 °C and 60 rpm. Results are presented as mean ± SD (n = 3). Statistical significance was determined using two-way ANOVA with Tukey’s post hoc test (*p < 0.05, **p < 0.01, ***p < 0.001, ***p < 0.0001).

**FIGURE 11:**
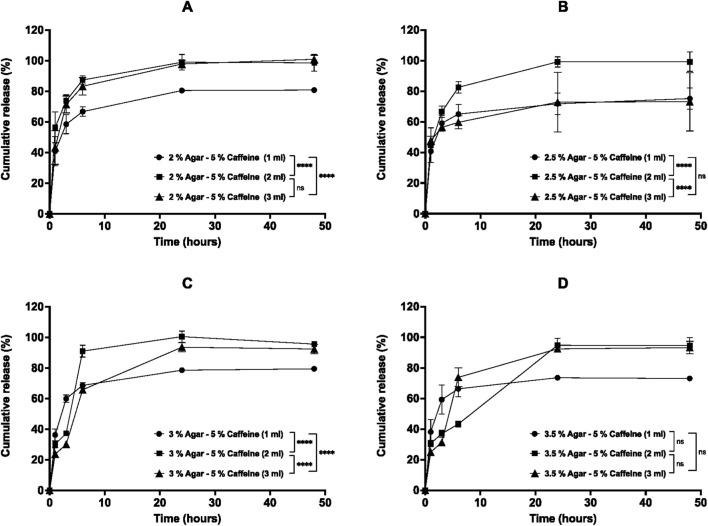
*In vitro* cumulative release profiles of 5% caffeine post-loaded at different volumes (1, 2, and 3 mL) into agar aerogels: **(A)** 2%, **(B)** 2.5%, **(C)** 3%, and **(D)** 3.5% agar concentrations in PBS (pH 7.4) over 48 h at 37 °C and 60 rpm. Results are presented as mean ± SD (n = 3). Statistical significance was determined using two-way ANOVA with Tukey’s post hoc test (*p < 0.05, **p < 0.01, ***p < 0.001, ***p < 0.0001).

**FIGURE 12:**
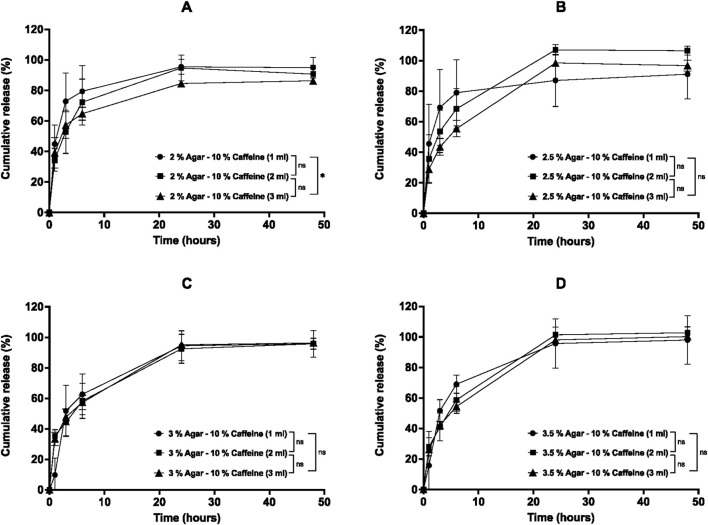
*In vitro* cumulative release profiles of 10% caffeine post-loaded at different volumes (1, 2, and 3 mL) into agar aerogels: **(A)** 2%, **(B)** 2.5%, **(C)** 3%, and **(D)** 3.5% agar concentrations in PBS (pH 7.4) over 48 h at 37 °C and 60 rpm. Results are presented as mean ± SD (n = 3). Statistical significance was determined using two-way ANOVA with Tukey’s post hoc test (*p < 0.05, **p < 0.01, ***p < 0.001, ***p < 0.0001).

The percentage cumulative release profiles for caffeine post-loaded aerogels are shown in Figures 10–12. The release of caffeine from the post-loaded aerogels is controlled by three factors, including the agar concentration of the aerogels, the caffeine loading concentration, and post loading volume. Similarly, the caffeine displayed a biphasic release profile over 48 h from post-loaded aerogels. First, a rapid ‘burst’ release, then the second phase of release that yielded a slower release after 3-6 h as the caffeine loading, and agar concentration increased. First, the effect of post-loading volume (1, 2, and 3 mL) of 1% caffeine into all aerogels on the release profiles are illustrated in Figure 10. Results demonstrated that the release profiles from post-loaded aerogels were relatively similar, and the caffeine was released rapidly during 6 h from aerogels until approaching complete release after 48 h for 1 and 2 mL post-loaded caffeine. However, aerogels post-loaded with 3 mL of 1% caffeine had a statistically significant (*p* < 0.05) slower release profile compared to 1 and 2 mL within most aerogel formulations, as well as with increasing the aerogels agar concentrations in 2, 3, and 3.5% aerogels mainly at 24 and 48 h. The release profiles of different post-loadings (1, 2, 3 mL) of 1% caffeine from 2% agar aerogel at different time points were compared. Within 6 hours, 2% agar aerogel post-loaded with 1 and 3 mL caffeine showed relatively similar release profiles, while 2 mL post-loaded caffeine demonstrated faster release mainly at 3 and 6 h. However, 3 mL post-loaded caffeine showed significantly (*p* < 0.05) slower release compared to 1 and 2 mL (Figure 10A). The release profiles of different post-loadings (1, 2, 3 mL) of 1% caffeine from 2.5% agar aerogel at different time points were compared. Within 6 hours, 1 mL post-loaded caffeine showed lower release compared to 2 and 3 mL, while non-significant (*p* > 0.05) differences in the release profiles between 2 and 3 mL post-loaded caffeine were observed (Figure 10B). The release profiles from 3% agar aerogel showed statistically non-significant (*p* > 0.05) difference between 1 and 2 mL post-loaded caffeine, while 3 mL post-loaded caffeine demonstrated significant (*p* < 0.05) slower release profile compared to 1 and 2 mL post-loaded caffeine (Figure 10C). The release profiles from 3.5% agar aerogel showed statistically significant (*p* < 0.05) difference between 1, 2, and 3 mL post-loaded caffeine with slower release profile as the caffeine post-loading increase (Figure 10D). Most of the 1% caffeine content was released after 48 h from all post-loaded aerogels. Upon post-loading similar volumes of 5% caffeine into aerogels, they provided different release from different aerogels compared to previous 1% post loaded aerogels as described in Figure 11. Results showed that 1 mL of 5% caffeine provided a relatively equal initial burst release profiles until 3 h followed by extended slow sustained release from all the formulations regardless the increased aerogels agar concentrations. At 48 h, post-loaded aerogels 2, 2.5, 3, and 3.5% released a total of 80.7 ± 1.6, 75.2 ± 6.9, 79.5 ± 1.5, and 73.1% ± 1%, respectively of their 1 mL 5% caffeine contents. In contrast, aerogels loaded with 2 mL of 5% caffeine displayed significantly faster release by rapidly releasing the majority of their caffeine within 6 h except from 3.5% aerogel that released only 43.2% ± 2% at 6 h (Figure 11D). Subsequently, all aerogels released most of their 2 mL 5% caffeine content after 48 h. The release from aerogels post-loaded with 3 mL of 5% caffeine were varied between the formulations. 2% aerogel exhibited the fastest release of its 3 mL 5% caffeine by releasing 83.3% ± 5.7% at 6 h until releasing 97.8% ± 2.5% after 48 h (Figure 11A). In contrast, results in Figure 11 B presented a slower sustained release of the 3 mL caffeine from 2.5% aerogel (59.6% ± 4.2%) at 6 h up to 73.1% ± 19% after 48 h. The additional increase in aerogels agar concentrations (3% and 3.5%) reduced the burst release of 3 mL caffeine contents in both formulations, followed by rapid increase in the release until releasing 92.4% ± 3% and 93.3% ± 4%, respectively after 48 h (Figures 11C,D). The release of 1 mL post-loaded 5% caffeine from every aerogel formulation was significantly slower (*p* < 0.05) compared to 2 mL in 2, 2.5, and 3% post-loaded agar aerogels, as well as to 3 mL in 2% and 3% post-loaded agar aerogels over 48 h. The release profiles of 1, 2, and 3 mL 10% caffeine from agar aerogels 2, 2.5, 3, and 3.5% are illustrated in Figure 12. Results demonstrate that the effect of different post-loading volumes is limited, as no significant (*p* > 0.05) differences were found in the release profiles of different caffeine volumes in the same aerogel formulation except between 1 and 3 mL in 2% post-loaded agar aerogel. However, the increased agar aerogels concentrations have the superior role over the release profiles rates of caffeine. Aerogels with higher agar concentrations improved the sustained release within 6 h, and subsequently most aerogels released their caffeine contents after 48 h. Key kinetic parameters for post-loaded aerogels, including burst release (6 h), time to 50% release (t_50_%), and Korsmeyer–Peppas exponent (n), are summarized in Table 4. All post-loaded formulations exhibited rapid initial diffusion with n values between 0.44 and 0.71, consistent with Fickian or near-Fickian release behavior.

**TABLE 4:** Summary of release kinetics for post-loaded agar aerogels.

Caffeine (%)	Volume mL	Burst at 6 h (% of M ∞ )	t_50_% (h)	K–P (n)	R^2^
**1%**	1 mL	70.8	0.78	0.523	0.999
	2 mL	81.4	0.65	0.514	0.997
	3 mL	80.4	0.64	0.438	0.998
**5%**	1 mL	82.6	0.60	0.563	0.999
	2 mL	88.6	0.48	0.709	0.999
	3 mL	82.6	0.58	0.594	0.999
**10%**	1 mL	83.7	0.60	0.643	0.999
	2 mL	79.7	0.69	0.612	0.999
	3 mL	75.0	0.77	0.578	0.999

Bold values indicate the most relevant kinetic parameters for comparison, highlighting formulations with different loading concentrations and its effect on slower sustained release and higher diffusion control.

## 4 Discussion

Agar aerogels were investigated for their potential as an implantable drug delivery system. Agar aerogels were loaded with caffeine using two different methods. First, the conventional gelation process which is commonly known as the sol-gel method, followed by solvent extraction using freeze-drying or known as lyophilization technique. Several preloaded aerogel formulations were prepared with increased agar (2, 2.5, 3, and 3.5% w/v) and caffeine (1, 5% and 10% w/v) loadings. Second, aerogels were prepared using the same sol-gel, freeze-drying methods and agar concentrations. Then, aerogels were post-loaded with caffeine. Different post-loaded aerogels were prepared with increased agar (2, 2.5, 3, and 3.5%) and increased caffeine loadings (1, 2, and 3 mL) of every caffeine concentration (1, 5, and 10% w/v). The aerogels were characterised for strength, porosity, swelling, drug content, as well as *in vitro* drug release.

The development of aerogels as a drug delivery system with appropriate mechanical properties and drug release profiles is of importance to achieve the required optimal therapeutic outcomes (Huang et al., 2011; Matricardi et al., 2013; Yuan et al., 2018). In our study, the mechanical strength investigations conducted on agar aerogels showed a direct relationship between the agar concentration and their increased mechanical strength. Accordingly, the aerogels prepared using high agar concentration (3.5%) produced aerogels with improved mechanical properties, while those aerogels with a reduced mechanical strength were associated with a decreased agar concentration (2%). The improved mechanical properties associated with the higher agar concentrations resulted from the higher rates of crosslinking during the gelation process due to the increased hydrogen bonds between the interconnected agar chains. Correspondingly, thicker and stronger network that provided better mechanical features of the aerogel (Deszczynski et al., 2003; Kin and Yaphe, 1972). However, the aerogels showed lower strength and more flexibility compared to the hydrogels due to the freeze-drying solvent extraction.

The lyophilization phase in agar hydrogel drying eliminates the liquid/vapor interface inside the pores, avoiding the formation of capillary pressure, which collapses the network. Consequently, without surface tension forces applied on the skeletal framework, hydrogel freeze drying process leaves the geometric dimensions of the water filled gaps unaffected. This resulted in low-density aerogel network structure with high porosity and flexibility (Li et al., 2009).

The caffeine increased loading into the aerogels significantly increased their physical strength, mainly at higher loadings. This is attributed to the increased caffeine accumulation with higher caffeine loadings within the aerogels network pores. This may results in higher aerogels density compared to the free aerogels, and consequently increased mechanical strength (Buckley et al., 2009).

In addition, the investigation of post-loading method into aerogels produced aerogels with significantly (*p* < 0.05) lower mechanical properties compared to free aerogels regardless their agar concentrations. Results have shown that the measured strength of the aerogels is significantly deteriorating upon injecting higher volumes into all aerogels. The reduction in the mechanical properties of post-loaded aerogels is mainly attributed to the rehydration of the aerogels network chains that corresponds with the disintegration of network structure and subsequently aerogel collapse (Fabra et al., 2021; Nweke et al., 2017). As a result, caffeine preloaded agar aerogels offer good mechanical strength based on the agar concentration and caffeine loading rate. Such improved mechanical properties are beneficial in the development of an implantable preloaded aerogels as a localised drug delivery system. This will provide support and resistance to damage upon implantation. However, the post-loaded aerogels lack the increased mechanical features compared to preloaded aerogels under physical stress.

In comparison to agar hydrogels, their freeze-dried aerogels may offer better access to core region and more efficient interactions with the aerogel matrix. Nevertheless, adjusting the aerogels polymeric components and the rate of crosslinking, could influence aerogels’ ability to control drug release or, in contrast, their ability as drug diffusing enhancers (Valo et al., 2013; Alnaief et al., 2012; Ulker and Erkey, 2014; Veronovski et al., 2013). In addition, the adjustable porous structure and mechanical integrity of agar aerogels highlight their potential as controlled drug delivery platforms. Porosity directly influences the release profile, while mechanical strength determines structural stability. Both parameters are critical for implantable systems. Also, aerogels could offer the option for drug loading method to be either preloaded during the formulation preparation or post-loaded after obtaining and implanting the formulation. The post loading method may offer better flexibility for the surgical team to load the required drugs and dose upon implantation.

The porosity and swelling ratios of the agar aerogels were investigated to identify the effect of increased agar concentration on aerogels porous structures and water retaining capabilities. Such features could influence their physical strength and drug release profiles from aerogel formulations (Pantić et al., 2020; Lázár et al., 2020; Veres et al., 2017). Results showed that the aerogel porosity ratios were decreased as the agar concentration increased. The significant (*p* < 0.05) reduction in porosity was recorded between aerogels with increased agar concentration by at least 1%. There was no statistical significance found (*p* > 0.05) between aerogels upon increasing the agar concentrations by 0.5%. The reduction in the aerogels porosity ratios is mainly due to the higher nucleation rate and closer chain packing of the aerogel network associated with increasing the agar concentration (Narayanan et al., 2006). Similarly, agar aerogels denoted non-significant (*p* > 0.05) reduction in their swelling rate while increasing agar concentration by 0.5% from 2 to 2.5% and/or 3%–3.5%, but the significance (*p* > 0.05) was noticed while adjusting the agar concentration by at least 1%. This resulted from the higher aerogels crosslinking rates as the agar concentration increase. Also, the presence of additional accumulating non-anhydrous moieties of agaropectin (non-gelling component of agar), could prevent the formation of conductive network structure for water absorption at certain crosslinking sites and thus limiting the swelling of aerogels with higher agar concentrations (Bao et al., 2010; Pourjavadi et al., 2009; Bertasa et al., 2020).

The caffeine content and distribution investigations were performed on all agar aerogels (2, 2.5, 3, and 3.5%) preloaded with caffeine (1, 5, and 10%). Results demonstrated that there are non-significant (*p* > 0.05) differences between the aerogels caffeine contents as the drug loading and agar concentration increase. Generally, the aerogels with lower agar concentrations (2% and 2.5%) loaded with 1 and 5% caffeine demonstrated improved caffeine distribution between the aerogel different parts compared to higher agar aerogels (3% and 3.5%). However, most aerogels with higher caffeine loading (10%) showed variations in their caffeine content distributions between the different parts of the aerogel. The differences in content distribution can be related to the increased crosslinking density with higher agar concentrations, which will result in formation of more heterogenous network structure and consequently affecting drug homogeneity, especially with higher loadings (Liang et al., 2006). Also, high drug loading could affect drug homogeneity within aerogel formulations, and probably longer mixing time is required to obtain more homogenous dispersion due to the increased viscosity of the gel solutions. As a result, using lower agar concentrations (2% and 2.5%) and lower drug loadings (1% and 5%) are preferable to obtain preloaded aerogel with more homogenous drug distribution as an implantable drug delivery system.

The distribution of different post-loaded caffeine concentrations (1, 5, and 10%) in agar aerogels (2% and 2.5%) was investigated using Raman spectroscopy. Results illustrated that both agar aerogel samples with low caffeine loading (1%) demonstrated limited drug distribution, while better homogenous distribution was found in those with higher caffeine loadings (5% and 10%). This may suggest that post-loading method can be more efficient to achieve better homogeneity especially for higher drug loading rates. However, to develop homogenous agar aerogels as an implantable drug delivery system based on post-loading method, further investigations using higher agar concentrations, several volumes of different drug concentrations, and wider scanning regions are required to confirm these findings.

The release of caffeine from most preloaded aerogels showed a rapid ‘burst’ release during the first 6 h for all drug loadings. As the caffeine loadings and agar concentrations increase the release of caffeine reduces within the first 3-6 h with sustained release over the remaining 48 h. The influence of increased caffeine loading, and agar concentration on the release profiles from preloaded aerogels was observed. This is referred to the several steps required for the caffeine release following their placement the preloaded aerogels in the release media. First, the hydration phase of the aerogel outer surface and adsorbed caffeine, followed by gradual penetration through the aerogel pores. Then, hydration of the aerogel network and the loaded caffeine within the core of the aerogel leading to caffeine desorption, dissolution and release (García-González et al., 2021). As a result, the release is governed by diffusion into the aerogel matrix. This process depends on porosity and polymer concentration, which together determine overall drug transport behavior. These observations align with broader reports on polysaccharide aerogels as tunable drug delivery matrices, where porosity, polymer concentration, and drying conditions critically influence diffusion behavior and release kinetics (García-González et al., 2021; Ganesan et al., 2018). Therefore, the fast release from the aerogel is mainly referred to caffeine hydrophilicity in addition to many factors, including the presence of drug molecules on the outer surface of aerogels that will dissolve rapidly. Also, the large open pores of the aerogels with lower agar concentrations will allow faster diffusion of lower caffeine loading (1%) that require shorter time to hydrate and dissolve (Pekarek et al., 1993; Han et al., 2013; Brazel and Peppas, 1999). In contrast, the significant slower sustained release profiles from preloaded aerogels were related to the reduced network pore sizes in higher aerogels agar concentrations (3% and 3.5%) and higher caffeine loading (5% and 10%) simultaneously. Consequently, slower hydration rate of the aerogels and the loaded highly concentrated caffeine molecules inside the formulations resulting in slower release profiles. Overall, these findings suggest that the drug release from preloaded aerogels is adjustable based on varying the aerogels agar concentrations and drug loadings. Also, they are able to release sufficient drug amount after 2 days upon using 2 and 2.5% agar aerogels and drug loadings 1% and 5%, while longer time is needed for higher drug loading (10%). However, the extremely fast burst release behaviour of 1% loading from 2 to 2.5% agar aerogels could be associated with unwanted side effects. The 3 and 3.5% agar aerogels relatively reduced the burst release effect of low drug loading, while high drug loading (10%) possibly require longer release time up to 4 or 5 days.

The release profiles from caffeine post-loaded aerogels were assessed and showed different release profiles over 48 h. First, the increased volume of 1% caffeine post-loaded into the agar aerogels (2, 2.5, 3, and 3.5%) is directly related to the slower release from the aerogels. These results suggest that the release media were able to penetrate the aerogels and facilitate the release of different caffeine loadings successfully from the lower side in contact with the release media. Differently, 5% caffeine post-loaded aerogels expressed slow sustained release of 1 mL caffeine in all formulation regardless their agar components, which implies that the remaining caffeine could be entrapped inside the networks. Nevertheless, as the aerogels agar concentrations increased, they demonstrated relatively slower release profiles of higher post-loaded volumes (2 and 3 mL) mainly within 6 h of release. This occurred due to consecutive factors including the narrowed network pore sizes and the higher caffeine loadings that require longer time to be released. Upon loading different volumes of 10% caffeine, post-loaded aerogels (2% and 2.5%) demonstrated release profiles that are controlled by both increased volume loading and agar concentration. However, the increased agar concentrations in 3% and 3.5% post-loaded aerogels apparently have the principal role in reducing the release mainly within the first 6 h compared to 2 and 2.5% aerogels, since there is no statistical significance (*p* > 0.05) in the release profiles of varied volumes within the same aerogel. Generally, post-loaded agar aerogels demonstrated their capability to reduce the release of increased volumes (1, 2, and 3 mL) of increased caffeine concentrations (1, 5, and 10%) within 6 h as the agar concentration increased. However, the extended-release profiles from the aerogels become less efficient with higher caffeine concentration (5%) and low loading volume (1 mL) which may have resulted from insufficient drug distribution or the reduced mechanical strength of the aerogels. As a result, the post-loaded aerogels with higher agar concentration and increased loading volume are promising for further investigations to be developed as an implantable drug delivery system.

One of the technologies that should be considered in future investigations during agar aerogel preparation is based on cryoprotection against ice crystals formation within the formulation upon placing agar hydrogel in −80 °C freezer prior to the lyophilization process. During the cooling process, the water molecules start to aggregate and aligned to form hydrogen bonds resulting in ice crystals formation, which could influence the hydrogel/aerogel network in terms of structural design and physical properties that could affect aerogels strength and porosity. Consequently, this might have an impact on the drug distribution and related to the change in the hydrogel and preloaded aerogel formulations drug homogeneity with increased drug loading to 10% and agar concentration (3% and 3.5%). Also, the change in the network structure and porosity design resulting from the ice crystal formation could have an influence on the release profiles behaviour as seen in post-loaded agar aerogels mainly. Therefore, different cryoprotection techniques during hydrogel preparation were developed in order to maintain and improve aerogels mechanical integrity as well as network structural design and porosity. One of the suggested methods is based on developing high-strength and fast self-healing hydrogel with an interpenetrating double network based on polyvinyl alcohol/agar-ethylene glycol (PVA/agar-EG) (Han et al., 2020). The addition of, EG during hydrogel preparation as a cryoprotectant agent and as a solvent with water prevents ice crystals formation at extremely low temperatures due to the hydrogen bonds formed between, EG and H_2_O that bind water molecules, which make it difficult for water molecules to aggregate and bind to each other. As a result, more stable hydrogel properties can be obtained for aerogel development with improved physical and internal structure that may result in enhanced homogenous drug distribution, as well as better controlled release profiles even with increased agar concentration and drug loading to achieve better implantable drug delivery systems. However, further investigations are required to support and confirm that.

From a translational perspective, implantable biomaterial systems must comply with regulatory requirements concerning sterilization validation, biocompatibility (ISO 10993), and long-term stability, which will be important considerations in future development stages (International Organization for Standardization, 2018).

## 5 Limitations and future perspectives

While caffeine was selected as a model compound due to its chemical stability, high aqueous solubility, and ease of quantitative detection by HPLC, its small molecular size and neutral, hydrophilic character may not fully represent the behavior of larger, hydrophobic, or charged therapeutic agents such as chemotherapeutics or protein-based drugs. Future studies should therefore evaluate drug release behavior using molecules with varying molecular weights and physicochemical properties to better establish generalizability.

In addition, although agar is widely regarded as biocompatible, no *in vitro* cytocompatibility or inflammatory response studies were conducted in the present work. For translational applications involving implantation, systematic evaluation using relevant cell lines (e.g., fibroblasts or macrophages) will be necessary to confirm biological safety.

Furthermore, sterilization compatibility was not assessed. Implantable systems require validation under clinically relevant sterilization methods such as gamma irradiation or ethylene oxide treatment. Future investigations should examine whether such processes alter aerogel microstructure, mechanical integrity, or drug stability.

Finally, long-term storage stability was not evaluated. Assessing structural integrity and drug retention over extended storage periods under controlled environmental conditions will be essential for practical deployment and regulatory advancement.

## 6 Conclusion

This study successfully formulated and characterised caffeine-loaded agar aerogels using both preloading and post-loading approaches, demonstrating their promise as tunable implantable drug delivery platforms. By systematically varying agar concentration, caffeine loading, and loading method, the work establishes clear relationships between formulation parameters, mechanical integrity, porosity, drug distribution, and release behaviour.

Higher agar concentrations consistently enhanced mechanical strength and reduced porosity and swelling, yielding structurally robust aerogels suitable for implantation. Preloaded aerogels exhibited superior mechanical stability compared to post-loaded counterparts, which experienced structural weakening proportional to the injected loading volume. Drug distribution analysis revealed that lower agar concentrations (2%–2.5%) and moderate caffeine loadings (1%–5%) achieved the most homogeneous distribution, while higher agar concentrations and high drug loadings introduced network heterogeneity.

Across all formulations, caffeine release followed a biphasic pattern marked by an initial burst release followed by sustained diffusion. Importantly, both the magnitude of the burst phase and the duration of sustained release were strongly dependent on agar concentration, drug loading, and loading technique. Preloaded formulations with 5% caffeine and post-loaded aerogels loaded with 1% caffeine (1–3 mL) were identified as optimal candidates, offering balanced mechanical strength, controlled release, and adequate drug distribution.

Overall, this work highlights the versatility of agar aerogels as adaptable carriers capable of meeting diverse therapeutic requirements through simple modifications of formulation parameters. The identified optimal formulations warrant further investigation, including biocompatibility assessment, long-term stability testing, and evaluation with clinically relevant drugs. With additional refinement, agar-based aerogels represent a promising platform for the development of customizable, minimally invasive implantable drug delivery systems.

## Data Availability

The original contributions presented in the study are included in the article/supplementary material, further inquiries can be directed to the corresponding author.

## References

[B1] Alnaief M. Antonyuk S. Hentzschel C. Leopold C. Heinrich S. Smirnova I. (2012). A novel process for coating of silica aerogel microspheres for controlled drug release applications. Microporous Mesoporous Mater 160, 167–173. 10.1016/j.micromeso.2012.02.009

[B2] Bao X. Hayashi K. Li Y. Teramoto A. Abe K. (2010). Novel agarose and agar fibers: fabrication and characterization. Mater Lett. 64 (22), 2435–2437. 10.1016/j.matlet.2010.08.008

[B3] Barrios E. Fox D. Li Sip Y. Y. Catarata R. Calderon J. E. Azim N. (2019). Nanomaterials in advanced, high-performance aerogel composites: a review. Polymers 11 (4), 726. 10.3390/polym11040726 31010008 PMC6523290

[B4] Bertasa M. Dodero A. Alloisio M. Vicini S. Riedo C. Sansonetti A. (2020). Agar gel strength: a correlation study between chemical composition and rheological properties. Eur. Polym. J. 123, 109442. 10.1016/j.eurpolymj.2019.109442

[B5] Brazel C. S. Peppas N. A. (1999). Mechanisms of solute and drug transport in relaxing, swellable, hydrophilic glassy polymers. Polymer 40 (12), 3383–3398. 10.1016/s0032-3861(98)00546-1

[B6] Buckley C. T. Thorpe S. D. O’Brien F. J. Robinson A. J. Kelly D. J. (2009). The effect of concentration, thermal history and cell seeding density on the initial mechanical properties of agarose hydrogels. J. Mech. Behav. Biomed. Mater 2 (5), 512–521. 10.1016/j.jmbbm.2008.12.007 19627858

[B7] Deszczynski M. Kasapis S. MacNaughton W. Mitchell J. R. (2003). Effect of sugars on the mechanical and thermal properties of agarose gels. Food Hydrocoll. 17 (6), 793–799. 10.1016/s0268-005x(03)00100-0

[B8] Du A. Zhou B. Zhang Z. Shen J. (2013). A special material or a new state of matter: a review and reconsideration of the aerogel. Materials 6 (3), 941–968. 10.3390/ma6030941 28809350 PMC5525157

[B9] Fabra M. J. Talens-Perales D. Roman-Sarmiento A. López-Rubio A. Polaina J. (2021). Effect of biopolymer matrices on lactose hydrolysis by enzymatically active hydrogel and aerogels loaded with β-galactosidase nanoflowers. Food Hydrocoll. 111, 106220. 10.1016/j.foodhyd.2020.106220

[B10] Ganesan K. Budtova T. Ratke L. Gurikov P. Baudron V. Preibisch I. (2018). Review on the production of polysaccharide aerogel particles. Materials 11 (11), 2144. 10.3390/ma11112144 30384442 PMC6265924

[B11] García-González C. A. Sosnik A. Kalmár J. De Marco I. Erkey C. Concheiro A. (2021). Aerogels in drug delivery: from design to application. J. Control Release 332, 40–63. 10.1016/j.jconrel.2021.02.012 33600880

[B12] Han Y. Choi J. Kim H.-S. Kim H. Park J. (2013). Control of pore and window size of ceramic foams with tri-modal pore structure: influence of agar concentration. Mater Lett. 110, 256–259. 10.1016/j.matlet.2013.05.100

[B13] Han X. Li M. Fan Z. Zhang Y. Zhang H. Li Q. (2020). PVA/agar interpenetrating network hydrogel with fast healing, high strength, antifreeze, and water retention. Macromol. Chem. Phys. 221 (22), 2000237. 10.1002/macp.202070049

[B14] Huang D. Zuo Y. Zou Q. Zhang L. Li J. Cheng L. (2011). Antibacterial chitosan coating on nano-hydroxyapatite/polyamide66 porous bone scaffold for drug delivery. J. Biomater. Sci. Polym. Ed. 22 (7), 931–944. 10.1163/092050610X496576 20566065

[B15] International Organization for Standardization (2018). ISO 10993-1:2018. Biological evaluation of medical devices part 1: evaluation and testing within a risk management process. Geneva: ISO.

[B16] Kanokpanont S. Damrongsakkul S. Ratanavaraporn J. Aramwit P. (2012). An innovative bi-layered wound dressing made of silk and gelatin for accelerated wound healing. Int. J. Pharm. 436 (1), 141–153. 10.1016/j.ijpharm.2012.06.046 22771972

[B17] Kin N. Yaphe W. (1972). Properties of agar: parameters affecting gel-formation and the agarose-iodine reaction. Carbohydr. Res. 25, 379–385. 10.1016/s0008-6215(00)81648-1 4679708

[B18] Lázár I. Forgács A. Horváth A. Király G. Nagy G. Len A. (2020). Mechanism of hydration of biocompatible silica-casein aerogels probed by NMR and SANS reveal backbone rigidity. Appl. Surf. Sci. 531, 147232. 10.1016/j.apsusc.2020.147232

[B19] Li L. Yalcin B. Nguyen B. N. Meador M. A. B. Cakmak M. (2009). Flexible nanofiber-reinforced aerogel (xerogel) synthesis, manufacture, and characterization. ACS Appl. Mater Interfaces 1 (11), 2491–2501. 10.1021/am900451x 20356119

[B20] Liang S. Xu J. Weng L. Dai H. Zhang X. Zhang L. (2006). Protein diffusion in agarose hydrogel *in situ* measured by improved refractive index method. J. Control Release. 115 (2), 189–196. 10.1016/j.jconrel.2006.08.006 16996163

[B21] Liu Z. Ran Y. Xi J. Wang J. (2020). Polymeric hybrid aerogels and their biomedical applications. Soft Matter 16 (40), 9160–9175. 10.1039/d0sm01261k 32851389

[B22] Matricardi P. Di Meo C. Coviello T. Hennink W. E. Alhaique F. (2013). Interpenetrating polymer networks polysaccharide hydrogels for drug delivery and tissue engineering. Adv. Drug Deliv. Rev. 65 (9), 1172–1187. 10.1016/j.addr.2013.04.002 23603210

[B23] Narayanan J. Xiong J.-Y. Liu X.-Y. (2006). “Determination of agarose gel pore size: absorbance measurements vis a vis other techniques,” in JBphys conf ser: conference series (Bristol, United Kingdom: IOP Publishing).

[B24] Nita L. E. Ghilan A. Rusu A. G. Neamtu I. Chiriac A. P. (2020). New trends in bio-based aerogels. Pharmaceutics 12 (5), 449. 10.3390/pharmaceutics12050449 32414217 PMC7284463

[B25] Nweke M. C. Turmaine M. McCartney R. G. Bracewell D. G. (2017). Drying techniques for the visualisation of agarose‐based chromatography media by scanning electron microscopy. Biotechnol. J. 12 (3), 1600583. 10.1002/biot.201600583 28029739

[B26] Pantić M. Horvat G. Knez Ž. Novak Z. (2020). Preparation and characterization of chitosan-coated pectin aerogels: Curcumin case study. Molecules 25 (5), 1187. 10.3390/molecules25051187 32155739 PMC7179465

[B27] Pekarek K. J. Jacob J. S. Mathiowltz E. (1993). Double-walled microspheres for drug delivery. MRS Proc. 331 (1), 97–101. 10.1557/proc-331-97

[B28] Pourjavadi A. Farhadpour B. Seidi F. (2009). Synthesis and investigation of swelling behavior of new agar based superabsorbent hydrogel as a candidate for agrochemical delivery. J. Polym. Res. 16 (6), 655–665. 10.1007/s10965-009-9270-2

[B29] Ulker Z. Erkey C. (2014). An emerging platform for drug delivery: aerogel based systems. J. Control. Release 177, 51–63. 10.1016/j.jconrel.2013.12.033 24394377

[B30] Valo H. Arola S. Laaksonen P. Torkkeli M. Peltonen L. Linder M. B. (2013). Drug release from nanoparticles embedded in four different nanofibrillar cellulose aerogels. Eur. J. Pharm. Sci. 50 (1), 69–77. 10.1016/j.ejps.2013.02.023 23500041

[B31] Veres P. Kéri M. Bányai I. Lázár I. Fábián I. Domingo C. (2017). Mechanism of drug release from silica-gelatin aerogel—Relationship between matrix structure and release kinetics. Colloids Surf. B Biointerfaces 152, 229–237. 10.1016/j.colsurfb.2017.01.019 28113125

[B32] Veronovski A. Knez Ž. Novak Z. (2013). Preparation of multi-membrane alginate aerogels used for drug delivery. J. Supercrit. Fluids 79, 209–215. 10.1016/j.supflu.2013.01.025

[B33] Wei S. Ching Y. C. Chuah C. H. (2020). Synthesis of chitosan aerogels as promising carriers for drug delivery: a review. Carbohydr. Polym. 231, 115744. 10.1016/j.carbpol.2019.115744 31888854

[B34] Yang G. Xiao Z. Long H. Ma K. Zhang J. Ren X. (2018). Assessment of the characteristics and biocompatibility of gelatin sponge scaffolds prepared by various crosslinking methods. Sci. Rep. 8 (1), 1616. 10.1038/s41598-018-20006-y 29371676 PMC5785510

[B35] Yuan Y. Wang L. Mu R.-J. Gong J. Wang Y. Li Y. (2018). Effects of konjac glucomannan on the structure, properties, and drug release characteristics of agarose hydrogels. Carbohydr. Polym. 190, 196–203. 10.1016/j.carbpol.2018.02.049 29628238

[B36] Zhao D. Zhang X. Zhang Y. Xu E. Yan S. Xu H. (2024). Recent advances in the fabrication, characterization and application of starch-based materials for active food packaging: hydrogels and aerogels. Sustain Food Technol. 2 (3), 615–634. 10.1039/d4fb00030g

